# Measuring the functional impact of cognitive impairment in Huntington’s disease

**DOI:** 10.1007/s00415-021-10955-2

**Published:** 2022-01-21

**Authors:** Andrea Horta-Barba, Saül Martínez-Horta, Jesus Pérez-Pérez, Frederic Sampedro, Arnau Puig-Davi, Javier Pagonabarraga, Jaime Kulisevsky

**Affiliations:** 1 Movement Disorders Unit, Neurology Department, Hospital de la Santa Creu i Sant Pau, Mas Casanovas 90, 08041 Barcelona, Spain; 2 Department of Medicine, Universitat Autònoma de Barcelona (U.A.B.), Barcelona, Spain; 3 Institut d’Investigacions Biomèdiques-Sant Pau (IIB-Sant Pau), Barcelona, Spain; 4 Centro de Investigación en Red-Enfermedades Neurodegenerativas (CIBERNED), Madrid, Spain; 5 European Huntington’s Disease Network (EHDN), Ulm, Germany

**Keywords:** Huntington’s disease, Cognition, Functional assessment, Functionality, Cognitive impairment

## Abstract

**Background:**

Patients with Huntington’s disease (HD) exhibit a variable predominance of cognitive, behavioral and motor symptoms. A specific instrument focusing on the impact of cognitive impairment in HD over functional capacity is lacking.

**Objective:**

To address the need for a brief and specifically developed HD questionnaire able to capture functional aspects suspected to be sensitive to cognitive impairment.

**Methods:**

We developed and validated the “Huntington’s Disease-Cognitive Functional Rating Scale” (HD-CFRS) in 78 symptomatic carriers of the Huntington’s disease mutation. We also administered the HD-CFRS to a knowledgeable informant to measure the level of agreement. To explore the association between HD-CFRS scores and participants’ cognitive status, we administered objective measures of cognition. Participants were classified as cognitively preserved (HD-NC), as having mild cognitive impairment (HD-MCI), or as having dementia (HD-Dem).

**Results:**

The HD-CFRS showed concurrent validity and internal consistency in the three groups. HD carriers and informants in the HD-NC group obtained similar HD-CFRS scores. However, in patients with mild cognitive impairment and dementia, informers reported greater functional impairment than HD participants. The HD-CFRS total score showed strong correlations with measures assessing cognition.

**Conclusions:**

These findings support the utility of the HD-CFRS as a brief and reliable instrument to measure functional defects associated with cognitive impairment in HD. We believe this questionnaire could be a useful tool both for clinical practice and research.

## Introduction

Huntington’s disease (HD) is an autosomal-dominant monogenetic neurodegenerative disease caused by a cytosine–adenine–guanine (CAG) trinucleotide repeat expansion in the *HTT* gene [1]. Clinically, HD is characterized by a complex constellation of progressive motor abnormalities, cognitive decline and neuropsychiatric symptoms starting around mid-adulthood [2]. In association with the worsening of HD symptoms, all patients experience a progressive loss of functional independence [3]. The clinical stages of HD are defined on the basis of the degree of independence in functional capacity [4]. In HD, functionality is commonly addressed using the Total Functional Capacity (TFC) score and the Functional Assessment Scale (FAS) from the functional assessment of the Unified Huntington’s Disease Rating Scale (UHDRS) [5]. The TFC rates the level of functional independence in five domains: occupation, finances, domestic chores, activities of daily living (ADLs), and care. Each of these domains is rated based on the patient’s capacity to independently perform these activities. The FAS scale is based on 25 yes/no questions and qualifies abilities to independently perform ADLs. Given that both the TFC and the FAS provide a global measure of patients’ functional capacity, these instruments have been extensively used as outcome measures and classification parameters in clinical trials and in other research settings [6, 7].

Patients with HD present a variable predominance and severity of cognitive, motor, and behavioral symptoms, all of which may contribute to functional capacity in a different way. However, the TFC and the FAS do not distinguish between the independent contribution of each of these domains, or those of mixed domains, to patients’ global functionality. Further characterizing the influence of each domain on overall functioning may help routine clinical practice and research because changes in functional independence may be driven differently by motor, cognitive, or behavioral symptoms. Functional HD-specific instruments that can be used to assess the contribution of each of these symptoms are needed.

Progressive cognitive impairment is an essential feature of HD and the development of dementia is an inevitable consequence [8, 9]. Cognitive changes have an enormous impact on functional capacity in patients with neurodegenerative disease [10–12] or other conditions involving damage to the central nervous system damage [13]. Accordingly, instruments focusing on the impact of cognitive impairment on functional capacity have been developed in clinical contexts such as Parkinson’s disease, Alzheimer’s disease, and stroke [14–18]. Although functional assessment instruments in HD include items that can be influenced by cognitive changes, none of the instruments currently available is able to specifically isolate the impact of cognitive impairment on functionality. It was recently suggested that the Neuro-Qol cognitive measure questionnaire has psychometric properties[19] that are appropriate for HD and could reliably address cognitive status by assessing executive functions and general concerns [20, 21]. However, although it focuses on daily living, the Neuro-Qol is not exactly a cognitive-functional assessment instrument. It was not specifically developed for HD and it does not consider the influence of patients’ lack of awareness.

To overcome the lack of HD-specific cognitive-functional assessment instruments, we developed the “Huntington’s Disease-Cognitive Functional Rating Scale” (HD-CFRS). The approach we used was the one we used previously to develop a Parkinson’s disease-specific cognitive-functional assessment scale [14].

The HD-CFRS is a 5-min questionnaire specifically developed to capture a wide range of functional aspects considered to be affected by cognitive impairment but not by motor and neuropsychiatric symptoms. It is composed of 12 items that determine the degree of difficulty in performing activities of daily living involving dual tasking, sustained attention, planning and organization, problem solving, verbal expression, comprehension, temporal and spatial orientation, and memory.

The main aims of the present study were to study the psychometric properties of the HD-CFRS so as to provide the HD community with a specific screening method to capture the impact of cognitive changes on daily living functionality and an instrument of potential usefulness as a functional outcome measure in interventional trials.

## Methods

### Participants

We prospectively recruited 78 symptomatic, early-to-mild gene-mutation carriers (CAG > 39) from the HD outpatient clinic in the Movement Disorders Unit at Hospital de la Santa Creu i Sant Pau in Barcelona. Each gene-mutation carrier was accompanied by a knowledgeable informant (KI) such as their caregiver, partner, or family member. Exclusion criteria were presence of a neurological disorder other than HD, history of head trauma, epilepsy, drug abuse, non-corrected visual problems, active major psychotic or delusional syndrome, and severe language difficulties.

All patients and KI gave written informed consent. The study protocol was reviewed and approved by the local research ethics committee at Hospital de la Santa Creu i Sant Pau and the study was performed in compliance with the 1964 Declaration of Helsinki and latter amendments.

### Procedure

We recorded sociodemographic and clinical data, including age, education level, CAG repeat length, cognitive status and severity of neuropsychiatric symptoms. Each study visit included the administration of a battery of cognitive, behavioral, motor and functional assessments. Motor status and disease severity were assessed by a neurologist. Disease stage was determined according to the Shoulson and Fahn staging for HD [4]. Motor symptoms were rated using the UHDRS total motor score (UHDRS-TMS). The disease burden score (DBS) was calculated using the formula ([CAG—35.5] × age) [22]. Functionality was assessed with the UHDRS Functional Assessment Scale (FAS), the Independence scale (IS) and the TFC.

Behavioral symptoms were assessed using the short form of the Problem Behavior Assessment for HD (PBA-s) [23]. The PBA-s consists of a semi-structured interview designed to cover several neuropsychiatric symptoms occurring in HD by assessing their severity and frequency over the past 4 weeks. The PBA-s was administered simultaneously to both the HD carrier and the KI.

The screening measures used to assess global cognitive status were the Mini-Mental State Examination (MMSE) and the Parkinson’s Disease-Cognitive Rating Scale (PD-CRS). The PD-CRS has shown to be a reliable instrument to assess global cognition in HD, allowing patients to be classified into groups according to the severity of cognitive impairment [24]. The MMSE has been tested as a cognitive screening test for use in HD [25, 26], and despite, its limitations in terms of sensitivity to cognitive changes in this disorder, it provides measurements that are not covered by the PD-CRS and that may be relevant for the present study (i.e., temporal and spatial orientation). As additional cognitive measures, we administered the Stroop color-naming test, the word-reading and interference tests, the phonetic verbal fluency test with letters F, A and S (FAS), the semantic verbal fluency test (animals), parts A and B of the Trail Making Test (TMT), and the Symbol Digit Modalities Test (SDMT).

The HD-CRFS was administered to all participants and KI to measure the agreement between them. Because HD is associated with a significant pattern of lack of awareness about the severity and functional repercussion of symptoms [27, 28], we compared the HD-CRS total score provided by the KI to the total score provided by gene-mutation carriers. We also calculated the time it took to administer the HD-CFRS to both participants and KI. Global cognitive status, behavior and the HD-CFRS were assessed by a trained neuropsychologist, expert in HD.

To explore the discriminative capacity of the HD-CFRS to classify patients according to global cognitive status, we used an approach based on the combination of the Clinical Dementia Rating Scale (CDR) and the Independence Scale (IS) [24, 29]. The CDR is used to characterize cognitive and functional performance in memory, orientation, judgment and problem solving, community affairs, home and hobbies, and personal care. A CDR rated as 0 means absent cognitive impairment, 0.5 indicates questionable/very mild cognitive impairment, and ratings between 1 and 3 indicate the presence of mild-to-severe cognitive deficits. The IS scale is based on 25 yes/no questions and qualifies the ability to independently perform ADLs. Based on the answers provided, a score of “independence” is computed, ranging from 100% (no special care needed) to 10% (tube-fed, total bed care). A score above 80% means that the participant remains employed, does household chores, and manages finances. Accordingly, patients with a CDR score of 0 and IS score > 80% were classified as cognitively preserved (HD-NC), those with a CDR of 0.5 and IS score > 80% were classified as having mild cognitive impairment (HD-MCI), and those with a CDR ≥ 1 and IS score ≤ 80% were classified as having major cognitive impairment in the range of dementia (HD-Dem).

### HD-CFRS development and administration

The HD-CFRS is a semi-structured interview designed to measure cognitive-related functional changes in HD. It comprises 12 items, scored in a Likert-like manner, that have been specifically developed to take into account the prototypical neuropsychological characteristics of HD [8, 9, 30]. For the item development, expert members of an HD specialized unit met and generated items that were sufficiently sensitive to detect the most affected cognitive domains in HD. In addition, they examined the wording to make sure it was comprehensible. In a pre-testing stage, the interview was then randomly administered to patients visiting the HD unit. After receiving input from patients and informants, we modified the scale slightly to make it more understandable, and decided to administer it as a semi-structured interview. The items comprising the HD-CFRS assess dual tasking, sustained attention, organization, problem solving, language, orientation, and memory in daily living scenarios. Scoring is based on the frequency of having or not having some difficulty in performing the activities listed for each item (0 = none; 1 = some of the time; 2 = most of the time; 8 = subject has never done the activity in the past). When answers are scored “8”, this score is replaced by the mean score obtained in all the other items. The scale was developed and administered in Spanish. For the purposes of this manuscript, the HD-CFRS in Spanish was sent to an external language review service for translation into English (Appendix).

### Statistical analysis

Data are expressed as means ± standard deviations (SDs) for continuous variables and as percentages for categorical variables. Group differences were analyzed using analyses of variance (ANOVAs) and *t* tests for continuous variables, the Mann–Whitney test for ordinal data, and *X*^2^ for categorical variables. Normality analysis was carried out from frequency distribution and Kruskal–Wallis test was used for asymmetric variables. Internal consistency was assessed using Cronbach’s *α* coefficients and intraclass correlation coefficients (ICC, two-way mixed model and absolute agreement) were used to examine agreement between patients and caregivers regarding HD-CFRS scores. The association between HD-CFRS scores and the various cognitive measures was assessed using partial correlation coefficients, controlling for the effects of age, education, CAG, UHDRS-TMS, PBA-s and gender. Receiver-operating characteristic (ROC) curves were generated to determine the HD-CFRS cutoff scores that best discriminated between the three cognitive groups. Sensitivity, specificity, and positive (PPV) and negative predictive value (NPV) were calculated for the HD-CFRS cutoff points.

All statistical procedures were performed using SPSS software, and a *p* value < 0.05 was considered statistically significant.

## Results

### Clinical and sociodemographic data

The HD sample consisted of 78 symptomatic gene-mutation carriers (59% females, mean age 53.1 ± 10.9 years, mean CAG repeat length = 43.4 ± 2.8, mean years of education 11.2 ± 4.1). Twenty of the 78 were classified as HD-NC (mean age = 52.2 ± 9.5; mean CAG = 42.9 ± 2.4; mean years of education = 13.1 ± 3.7; mean TFC = 12.1 ± 1), 33 as HD-MCI (mean age = 51.7 ± 9.2; mean CAG = 43.1 ± 2.3; mean years of education = 11.2 ± 4.0; mean TFC = 10.6 ± 0.9) and 25 as HD-Dem (mean age = 55.6 ± 13.8; mean CAG = 44.0 ± 3.4; mean years of education = 9.9 ± 4.3; mean TFC = 7.0 ± 2.4).

Participants in the HD-NC were not significantly younger than HD-MCI [*t*(52) = 0.209; *p* = 0.835] or HD-Dem [*t*(44) = − 0.921; *p* = 0.362] participants. Furthermore, HD-NC participants had a similar level of education to the HD-MCI [*t*(52) = 1.634; *p* = 0.108] group. However, HD-NC had a significantly higher level of education than the HD-Dem group [*t*(44) = 2.516; *p* = 0.016]. As expected, functional capacity and motor values differed significantly between cognitive HD groups (see details in Table 1).

**Table 1:** Clinical and sociodemographic characteristics of the sample

	HD (*n* = 78)	HD-NC (*n* = 20)	HD-MCI (*n* = 33)	HD-Dem (*n* = 25)	*p*
Age	53.1 ± 10.9	52.2 ± 9.5	51.7 ± 9.2	55.6 ± 13.8	^a^0.835; ^b^0.362; ^c^0.203
Gender (f/m)	46/32	14/6	17/16	15/10	*χ*^2^ = ^a^0.150; ^b^0.352; ^c^0.354
Education	11.2 ± 4.1	13.1 ± 3.7	11.2 ± 4.0	9.9 ± 4.3	^a^0.108; ^b^0.016; ^c^0.249
CAG	43.4 ± 2.8	42.9 ± 2.4	43.1 ± 2.3	44.0 ± 3.4	^a^0.806; ^b^0.215; ^c^0.198
DBS^A^	396 ± 103.7	375.5 ± 97.1	375.7 ± 88.3	436.6 ± 117.4	^a^0.992; ^b^0.073; ^c^0.031
UHDRS-TMS^B^	38 ± 22.8	20.1 ± 16.3	34.6 ± 16.5	57.2 ± 21.4	^a^0.004; ^b^< 0.001; ^c^< 0.001
TFC^C^	9.8 ± 2.6	12.1 ± 1	10.6 ± 0.9	7.0 ± 2.4	^a^ < 0.001; ^b^< 0.001; ^c^< 0.001
IS	79.6 ± 12.6	92.9 ± 8.3	80.7 ± 7.6	67.9 ± 8.7	^a^ < 0.001; ^b^< 0.001; ^c^< 0.001
MMSE	24.9 ± 3.8	28.1 ± 1.4	25.8 ± 2.9	21.5 ± 3.5	^a^0.005; ^b^< 0.001; ^c^< 0.001
PD-CRS	73.5 ± 19.9	94.7 ± 11.1	75.7 ± 13.5	53.3 ± 9.1	^a^ < 0.001; ^b^< 0.001; ^c^< 0.001
HD-CFRS	5.6 ± 4	3.6 ± 2.8	5.2 ± 3.0	7.8 ± 4.9	^a^0.052; ^b^0.001; ^c^0.016
HD-CFRS (KI)	10.2 ± 6.5	3.2 ± 2.5	9.1 ± 3.2	17.2 ± 4.6	^a^ < 0.001; ^b^< 0.001; ^c^< 0.001
PBA-s					
Depression	3.4 ± 4.9	3.0 ± 3.3	3.8 ± 6.3	3.2 ± 3.9	^a^0.625; ^b^0.920; ^c^0.655
Irritability	3.5 ± 5.1	2.2 ± 3.2	4.7 ± 6.4	2.9 ± 4.1	^a^0.120; ^b^0.542; ^c^0.235
Apathy	4.6 ± 4.1	2.3 ± 3.0	4.4 ± 3.7	6.5 ± 4.3	^a^0.061; ^b^0.001; ^c^0.049
Psychosis	0.5 ± 1.9	0.4 ± 1	0.4 ± 1.3	0.6 ± 2.8	^a^0.930; ^b^0.757^; c^0.756
Executive dysfunction	3.7 ± 4.4	2.6 ± 3.6	3.1 ± 4.8	5.4 ± 4.2	^a^0.736; ^b^0.027^; c^0.065

^A^Disease burden score

^B^Unified Huntington’s disease rating scale-total motor score

^C^Total functional capacity

^a^HD-NC vs HD-MCI

^b^HD-NC vs HD-Dem

^c^HD-MCI vs HD-Dem

Focusing on behavioral variables, we found significant differences between HD-NC and HD-MCI and HD-Dem groups regarding apathy severity (see details in Table 1). Regarding global cognitive status, HD-MCI participants scored significantly lower than HD-NC in the PD-CRS [*t*(52) = 5.141; *p* < 0.001] and in the MMSE [*t*(52) = 2.960; *p* = 0.005]. HD-Dem participants scored significantly lower in the PD-CRS [*t*(57) = − 6.833; *p* < 0.001] and in the MMSE tests [*t*(57) = − 4.959; *p* < 0.001] than participants in HD-MCI group (see details in Table 1).

Regarding the total score of the HD-CFRS, and as seen in Table 1, HD-MCI almost had a significantly higher score than HD-NC in the HD-CFRS total score [*t*(52) = − 1.991; *p* = 0.052] when the HD-CFRS was answered by the participant. When it was answered by KI, the differences between HD-MCI and HD-NC groups were significant [*t*(52) = − 6.935; *p* < 0.001]. HD-Dem scored significantly higher than HD-MCI in the HD-CFRS total score corresponding to the participant [*t*(58) = 2.474; *p* = 0.016] and when answered by KI [*t*(58) = 7.874; *p* < 0.001].

The average time needed for the researcher to complete the HD-CFRS together with a semi-structured interview was 8 ± 2 min for HD participants and 5 ± 1 min for KI.

### Agreement between HD participants and KI in HD-CFRS

Looking at the whole HD sample, we found significant differences between answers provided by participants and KI [*t*(78) = − 7.232; *p* < 0.001]. Total scores in the HD-CFRS were lower for HD participants than for KI.

Focusing on the HD-NC group, we found no significant differences between answers provided by participants and those by KI regarding the HD-CRS total score [*t*(20) = − 0.972; *p* = 0.343]. The average ICC was 0.927 with a 95% confidence interval ranging from 0.82 to 0.971 [*F*(1,19) = 13.735, *p* < 0.001]. Correlation analysis between the HD-CFRS total score provided by participants and KI showed a significantly positive association (*r* = 0.870; *p* < 0.001). These associations remained strongly significant when age, education, CAG, UHDRS-TMS, PBA-s and gender were controlled in the partial correlation analysis (*r* = 0.802; *p* = 0.002).

Focusing on the HD-MCI group, we found significant differences when comparing HD-CFRS scores provided by participants and those provided by KI [*t*(33) = − 6.026; *p* < 0.001]. The average ICC was 0.463 with a 95% confidence interval ranging from − 0.087 to 0.735 [*F*(1,32) = 1.863, *p* = 0.058]. Correlation analysis between the HD-CFRS total score provided by participants and KI did not show a significant positive association (*r* = 0.302; *p* = 0.088). These associations were worse when age, education, CAG, UHDRS-TMS, PBA-s and gender were controlled in the partial correlation analysis (*r* = 0.323; *p* = 0.107).

Focusing on the HD-Dem group, we observed significant differences when comparing HD-CFRS scores between gene-mutation carriers and KI [*t*(25) = − 7.875; *p* < 0.001]. The average ICC was 0.354 with a 95% confidence interval ranging from − 0.466 to 0.715 (*F*(1,24) = 1.548, *p* = 0.146). Correlation analysis between the HD-CFRS total score provided by participants and KI did not show a significant positive association (*r* = 0.215; *p* = 0.301). These associations were worse when age, education, CAG, UHDRS-TMS, PBA-s and gender were controlled in the partial correlation analysis (*r* = − 0.041; *p* = 0.869).

### Correlations between HD-CFRS and cognitive measures

Partial bivariate correlation analysis was performed between HD-CFRS and global cognitive measures. Responses provided by HD participants showed a significant association between the HD-CRS and the total PD-CRS scores (*r* = − 0.336; *p* = 0.004), and the MMSE total score (*r* = − 0.245; *p* < 0.044). When answers were provided by the KI, strong correlations were found between the HD-CFRS total score and the PD-CRS total score (*r* = − 0.753; *p* < 0.001), and the MMSE total score (*r* = − 0.722; *p* < 0.001). Partial correlation coefficients controlling for age, education, CAG, UHDRS-TMS, PBA-s and gender showed mild-to-strong correlations between the HD-CFRS answered by KI and the PD-CRS total score (*r* = − 0.560; *p* < 0.001), and the MMSE total score (*r* = − 0.631; *p* < 0.001). When covariables were added, no significant association was found between the HD-CRS answered by HD participants and cognitive measures.

Based on the findings regarding the discrepancies between scores provided by KI and scores provided by gene-mutations carriers, we performed the following correlation analyses using the HD-CFRS total score provided by KI.

In the HD-NC group, no significant association was found between the HD-CFRS and disease stage (*r* = − 0.287; *p* = 0.233). In the HD-NC group, the association with the TFC was not significant (*r* = − 0.393; *p* = 0.096). In the HD-MCI group, no association was found between the HD-CFRS and the disease stage (*r* = − 0.201; *p* = 0.357) or with the TFC (*r* = 0.235; *p* = 0.281). In addition, in the HD-Dem, significant associations were found between HD-CFRS and the disease stage (*r* = 0.666; *p* = 0.004), and also between HD-CFRS and the TFC (*r* = − 0.593; *p* = 0.012).

The HD-CFRS showed significant associations with several subscores for all the cognitive measures obtained. All these associations remained significant after controlling for the effects of age, education, CAG, UHDRS-TMS, PBA-s and gender. In the MMSE, associations were found between the HD-CFRS total sore and orientation (*r* = − 0.532; *p* < 0.001), attention (*r* = − 0.503; *p* < 0.001), recall (*r* = − 0.319; *p* < 0.01), writing (*r* = − 0.391; *p* = 0.005), and copy of a pentagon (*r* = − 0.458; *p* = 0.001). In the PD-CRS, associations were found with immediate recall (*r* = − 0.264; *p* < 0.05), naming (*r* = − 0.290; *p* < 0.05), attention (*r* = − 0.455; *p* = 0.001), working memory (*r* = − 0.530; *p* < 0.001), drawing of a clock (*r* = − 0.418; *p* < 0.005), copy of a clock (*r* = − 0.318; *p* < 0.05), delayed recall (*r* = − 0.264; *p* < 0.05), alternate fluency (*r* = − 0.472; *p* < 0.001), fronto-subcortical score (*r* = − 0.521; *p* < 0.001), and posterior-cortical score (*r* = − 0.350; *p* < 0.05). In the other cognitive measures, associations were found with the SDMT (*r* = − 0.485; *p* < 0.001), semantic fluency (*r* = − 0.416; *p* = 0.002), Stroop color-naming (*r* = − 0.397; *p* < 0.05), Stroop word-reading (*r* = − 0.299; *p* < 0.05), Stroop interference test (*r* = − 0.519; *p* = 0.001), TMT-A (*r* = 0.392; *p* < 0.01), TMT-B (*r* = 0.516; *p* < 0.001) and verbal fluency (*r* = − 0.446; *p* = 0.001).

### Discriminative capacity of HD-CFRS

**Fig. 1:**
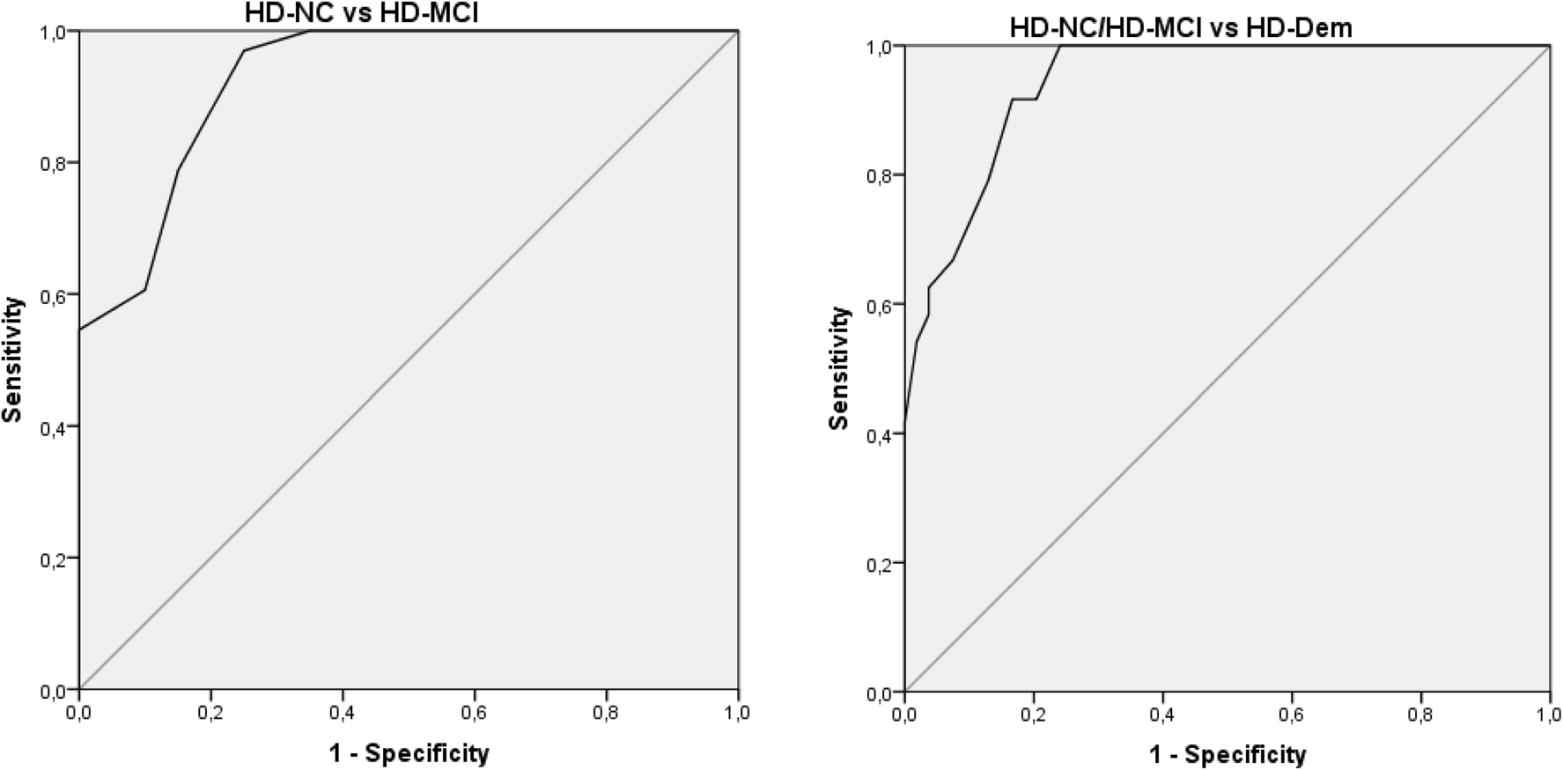
Receiver-operating characteristic (ROC) curves illustrating the discriminative properties of the HD-CFRS (KI)

Discriminant ROC analysis showed that a HD-CFRS total score ≥ 5.5/6.5 was the optimal cutoff to discriminate between HD-NC and HD-MCI [sensitivity, 88%; specificity, 80%; PPV, 93%; NPV, 86%; area under the curve (AUC) = 0.929; 95% CI 0.859–0.999]. A HD-CFRS total score ≥ 11.5/12.5 was the optimal cutoff to discriminate between HD-Dem and HD-MCI/NC [sensitivity, 92%; specificity, 83%; PPV, 92%; NPV, 68%; area under the curve (AUC) = 0.944; 95% CI 0.899–0.989] (Fig. 1; Table 2).

**Table 2:** Accuracy measures for the screening of MCI and dementia using different HD-CFRS (KI) cutoff scores

HD-CFRS cutoff for MCI	Sensitivity	Specificity
3.5/4.5	0.97	0.75
4.5/5.5	0.88	0.80
**5.5/6.5**	**0.79**	**0.85**

HD-CFRS cutoff for dementia	Sensitivity	Specificity
10.5/11.5	0.92	0.79
**11.5/12.5**	**0.92**	**0.83**
12.5/13.5	0.79	0.87

## Discussion

In the present study, we developed a brief HD-specific cognitive-functional assessment instrument and explored its main psychometric properties. We found it had good psychometric attributes and was sensitive to discriminate between patients with varying degrees of cognitive impairment.

The highly significant differences between scores rated by a symptomatic HD participant with mild-to-severe cognitive impairment and scores rated by KI suggest that cognitive-functional assessment in HD should focus on an interview with a reliable informant.

The HD-CFRS appeared to capture the functional impact of cognitive defects in the HD population. Specific cutoff scores were determined to detect patients with cognitive-functional deficits in the range of mild cognitive impairment (HD-CFRS cutoff ≥ 5.5/6.5), and in the range of dementia (HD-CFRS cutoff ≥ 11.5/12.5).

Our results also confirmed a strong association between cognitive impairment and functional alterations in HD, and showed that these functional alterations can be appropriately measured with the HD-CFRS. We also found correlations with several cognitive measures of sustained attention, cognitive flexibility, working memory, dual tasking, and processing speed. These cognitive alterations have been characteristically associated with the frontal-striatal damage typical of HD disease. The associations found thus highlight the functional impact of deficits at the levels of these processes. We also observed associations in items related to orientation, semantic integrity, writing, memory, and constructional praxis.

This study has some limitations. First, the scale was not developed together with an expert panel. It was guided by the review of the literature in the field and input from a group of experts working together in an HD unit. The purpose was to illustrate the usefulness of an instrument focused on the cognitive-functional aspects of HD. However, the scale will require revision; additional items should be explored, a 5-point or 7-point rating could be used for the rating scale format, and consensus by specialists should be reached. Therefore, as both Spanish and English versions of the HD-CFRS will require further validation they are not yet ready for use in clinical practice or research. Second, future studies with larger samples are needed as the sample sizes after group stratification were relatively small. Third, language and communication should be assessed in greater detail*.* Fourth, a KI is needed. Finally, longitudinal studies should be performed to demonstrate the ability of the HD-CFRS to detect functional changes over time.

Despite these shortcomings, the study has two main strengths. Above all, it is the first attempt to develop an HD-specific cognitive-functional rating scale. Furthermore, it can be completed in a short amount of time, usually taking no more than 5 min. It is also of note that we found appropriate psychometric attributes in terms of discriminative capacity and that we determined specific cutoffs to detect patients with cognitive-functional deficits in the range of mild cognitive impairment and in the range of dementia. The HD-CFRS could be considered for use both in clinical practice and for the design of interventional trials. Since functionality is a primary endpoint in interventional trials and because cognitive aspects of HD are a main target of these trials, an instrument able to measure specific cognitive-functional aspects of the disease is of major interest.

## Conclusions

The HD-CFRS appears to be a valid and reliable instrument to measure cognitive-functional changes in HD. We believe this questionnaire can be useful both in clinical and research contexts.

## Data Availability

Data supporting the findings in this study are available on request from the corresponding author. Such data are not publicly available due to privacy or ethical restrictions.

## Appendix

**Table:** Huntington’s disease cognitive functional rating scale (HD-CFRS)

		Score			
1	Do you have difficulties performing two tasks at the same time? (such as chatting while walking, or listening to someone while doing something else)	0	1	2	8
2	Do you have difficulties concentrating or paying attention? (e.g., following the plot of a movie or a book, or the thread of a conversation)	0	1	2	8
3	Do you have difficulties organizing what you have to do throughout the day? (and at the end of the day you have not done all the things you intended to)	0	1	2	8
4	Do you have difficulties planning or organizing your vacations or get-togethers with your family or friends?	0	1	2	8
5	Do you have difficulties controlling your correspondence, doctor visits, or times when to take medication, etc.?	0	1	2	8
6	Do you have trouble solving unforeseen or unexpected problems?	0	1	2	8
7	Do you have a hard time explaining what you want to say?	0	1	2	8
8	Do you have difficulties understanding what you read: books, magazines, newspapers?	0	1	2	8
9	Do you have a hard time remembering what day it is?	0	1	2	8
10	Do you have difficulties using public transport because it is difficult for you to understand which line to take?	0	1	2	8
11	Do you have difficulties finding your way in unfamiliar places?	0	1	2	8
12	Do you have a hard time remembering the things you have to do?	0	1	2	8
0: Never or almost never 1: Some of the time 2: Most of the time 8: Subject has never done the activity in the past		Total score: ___ (0–24)			
